# Characteristics of preplan‐based three‐dimensional individual template‐guided brachytherapy compared to freehand implantation

**DOI:** 10.1002/acm2.13840

**Published:** 2022-11-09

**Authors:** Bin Zhang, Siyu Zhang, Li Sun, Yaqin Wu, Yongqin Yang

**Affiliations:** ^1^ Jiangsu Cancer Hospital The Affiliated Cancer Hospital of Nanjing Medical University Jiangsu Institute of Cancer Research Nanjing China

**Keywords:** 3D‐printed template, freehand implantation, intracavitary/interstitial brachytherapy, locally advanced cervical cancer, preplan

## Abstract

Image‐guided adaptive intracavitary/interstitial brachytherapy (IC/IS IGABT) has exhibited superior dosimetry advantage and local control for locally advanced cervical cancer (LACC). Our group designed a type of cylindrical three‐dimensional (3D) printed vaginal template combining an intracavitary applicator with straight and oblique interstitial needles according to the preplan on computed tomography images. This work aimed to research the consistency of the preplan with the treatment plan at every fraction to verify the practical guiding significance of the preplan. We also investigated the difference between 3D‐printed template‐guided implantation compared with freehand implantation for LACC. Twenty‐six patients were treated with 3D‐printed individual templates (3D template group), and 20 patients were treated by using freehand insertion (freehand group). Patients in the 3D template group would take a preplan one week before treatment to design and print the individual template, while the freehand group did not. All patients accepted volumetric rotational intensity‐modulated radiotherapy at a dose of 49.4 Gy in 26 fractions and subsequent brachytherapy at a dose of 26 Gy in four fractions. All analyses were performed by utilizing SPSS 26. The insertion depth was decreased in fractions 1 and 4 compared with the preplan. None of the dose volume histogram parameters of fractions 1–3, nor the D_2cc_ of bladder and bowel at fraction 4 were barely changed compared with the preplan. The D_90_ and D_98_ of the high‐risk clinical target volume in the 3D template group were statistically higher than those in the freehand group (*p* < 0.01). The D_2cc_ of the rectum, bladder, bowel, and sigmoid in the 3D template group were all lower than those in the freehand group (*p* < 0.01). The preplan in this research is consistent with treatment plans, which is important to ensure the feasibility of applying a 3D‐printed template in brachytherapy. The 3D‐printed individual guidance template was an effective method in brachytherapy for locally advanced cervical cancer.

## 1 INTRODUCTION

External beam radiotherapy (EBRT) combined with synchronous chemotherapy followed by intracavitary brachytherapy (ICBT) is the traditional standard strategy for locally advanced cervical cancer (LACC). Nowadays, image‐guided adaptive brachytherapy (IGABT) has gained considerable attention, as it can improve the therapeutic efficacy of tumor control without enhancing the obvious toxicity of organs at risk (OARs).^1^, ^2^, ^3^, ^4^, ^5^ However, the local control rate in LACC can fall off for the unfavorable isodose of the high‐risk clinical target volume (HR CTV) treated with ICBT only in the presence of characteristics such as large tumor volume, eccentric location, irregular shape, and parametrial or other site invasions.^6^ Based on previous studies, image‐guided adaptive intracavitary/interstitial brachytherapy (IC/IS IGABT) is superior in terms of dosimetry advantages and local control rate for the treatment of large tumors.^7^, ^8^, ^9^ Nevertheless, standard IC/IS applicators such as the Vienna and Utrecht have limitations in wide clinical applications because of the uniform size, fixed distance between each needle hole, and unsuitability for patients with parametrial infiltration. Freehand implantation is a choice when the standard applicators cannot be used; however, operation using this method mainly depends on the experience of the gynecologist and usually results in an unfavorable needle position.

Three‐dimensional (3D) printed template‐guided implantation is a newly emerging technology used in brachytherapy with the goal of individual therapy to achieve high doses and optimally sculpted isodose tailored to the target, as well as an enhanced therapeutic effect with minimal invasion.^10^, ^11^, ^12^, ^13^, ^14^, ^15^ Lindegaard et al.^10^ reported a case where a standard intracavitary applicator was applied combined with a 3D‐printed individualized ring to treat a large target in the cervix uteri and resulted in preferable dose distribution compared to that in a patient treated with a standard Vienna applicator. Logar et al.^13^ demonstrated this further using a 3D‐printed personalized applicator to implement IC/IS BT when they found that it was difficult to achieve ideal dose coverage for patients at stages IIIB and IVA treated with a standard applicator; their results showed an increase of 8–9 Gy in the HR CTV prescription. Sekii et al.^14^ reported two cases where a polygon orthogonal projection method was utilized to create a 3D‐printed template; this allowed better dose distribution and operation‐time reduction, decreasing patient discomfort. Li et al.^15^ investigated the use of a 3D‐printed template for the treatment of two patients at stage IVA with pure interstitial needles and found that the 3D‐printed template was efficient and potentially valuable for use in clinical practice. Preplanning is always essential and crucial to obtain a personalized 3D guidance template that can achieve the geometric description of inserted needles as well as the anticipated dose distribution in the target volume and OARs.^16^, ^17^ Huang et al.^18^ designed individual printed templates based on preplanning data that included ^125^I seed locations and needle pathways for low‐dose‐rate radiation in patients with head and neck cancers. Sethi et al.^19^ inversely designed and printed 3D cylindrical templates through a preplanning process used for the pure interstitial therapy of two patients with vaginal tumors. However, studies on the characteristics of preplanning and the difference between preplanning and clinical planning are sparse, and the widespread application of printed guidance templates remains limited. Our research group has designed a type of cylindrical 3D‐printed vaginal template combining an intracavitary applicator with straight and oblique interstitial needles, individually printed for each patient according to the virtual plan devised based on CT images. Here, we aimed to study the consistency of the virtual plan with the treatment plan at every fraction to investigate the feasibility and repeatability of template‐guided insertion and the difference between 3D‐printed template‐guided implantation with freehand implantation in LACC therapy.

## 2 METHODS

### 2.1 Patient data

Forty‐six patients with biopsy‐proven LACC between April 2020 and December 2021 at our department were consecutively included in this study (Table 1). Twenty‐six of those were treated with a 3D‐printed individual template (3D template group), and the other 20 patients were treated using freehand implantation technology (freehand group). Staging determination depended on a gynecological examination under general anesthesia completed by a gynecologist and a radiation oncologist based on the 2009 FIGO system. After undergoing volumetric rotational intensity‐modulated radiotherapy at a dose of 49.4 Gy in 26 fractions every workday, all patients were treated using a high‐dose ^192^Ir afterloading brachytherapy machine at a dose of 26 Gy in four fractions once a week. Review magnetic resonance imaging and gynecological examination were conducted for all patients 1 week before brachytherapy. Patients in the 3D template group underwent preplanning the next day to design and print the individual template, while patients in the freehand group did not undergo other operations. All but three patients were treated with synchronous chemotherapy. The research plan for the 3D‐printed individualized template was approved by the Ethics Committee of our hospital before therapy. All participants offered written informed consent before enrollment in the study.

**TABLE 1:** Patient characteristics. Numbers in brackets indicate the percentage

		**3D template group**	**Freehand group**
Number of cases		26	20
Age	years, mean ± SD*	51.8 ± 8.7	60.3 ± 8.3
Pathology	Sq^@^	20 (77)	17 (85)
	Adc/AdSq^£^	6 (23)	3 (15)
FIGO‐stage	IIB	4 (15)	5 (25)
	IIIB	15 (58)	14 (70)
	IVA	3 (12)	1 (5)
	IVB	4 (15)	0 (0)
Volume of HRCTV	cm^3^, mean ± SD*	43.10 ± 14.99	40.65 ± 11.25
Synchronous chemotherapy	Yes	25 (96)	18 (90)
	No	1 (4)	2 (10)
Number of needles	Mean [range]	4.9 [4–7]	3.1 [2–4]

* Standard deviation.

^@^ Squamous cell carcinoma.

^£^ Adenocarcinoma or adenosquamous cell carcinoma.

### 2.2 Preplan and creation of the template

Patients in the 3D template group were placed in the lithotomy position on a thermoplastic mold, and a urinary catheter was inserted to empty the bladder and later inject saline. Appropriate diameters of templates and the degree of intracavitary applicators were chosen and inserted in patients according to the length and narrow‐angle of the vagina. The template used in the preplan only has one channel for intracavity applicators rather than needle channels. A total of 100 ml saline was infused into the bladder prior to computed tomography (CT) scanning. Patients laid flat with legs straightened during the CT scanning, with a slice thickness of 3 mm from the superior border of the first lumbar spine to 5 cm below the vulva. After CT scanning, images were transmitted to Oncentra Brachy 4.6 (Elekta AB, Stockholm, Sweden), and the template and intracavitary applicator were removed from the patients as soon as possible. The HR CTV, bladder, sigmoid, rectum, and bowel were delineated by the gynecologic oncologists on the CT images according to the guidelines published by Groupe Européen de Curiethérapie and the European Society for Radiotherapy and Oncology.^20^, ^21^

First, we reconstructed the template and intracavitary applicators in the Oncentra system and then designed the virtual interstitial needles according to the topography of the HR CTV and OARs. The positions of the interstitial needles were adjusted using an iterative manual process with respect to the loading pattern rules and dose limits of our institution. There are several principles for the construction of virtual needles, described as follows: (1) the least possible number of oblique needles should be used; (2) the distance between each needle cannot be less than 5 mm on the transverse section; (3) the exit of the oblique needles on the template should remain close to the HR CTV and away from the OARs; (4) the entry points of all needles on the final plane of the template should be more than 3 mm apart from each other to ensure the installation of the limiting stopper used to control the insertion depth of the needles; (5) oblique interstitial needles should not intersect with parallel needles. Finally, until the preplan was accepted by the gynecologist, the insertion depth of the virtual needles in the tissue was measured and recorded.

**FIGURE 1:**
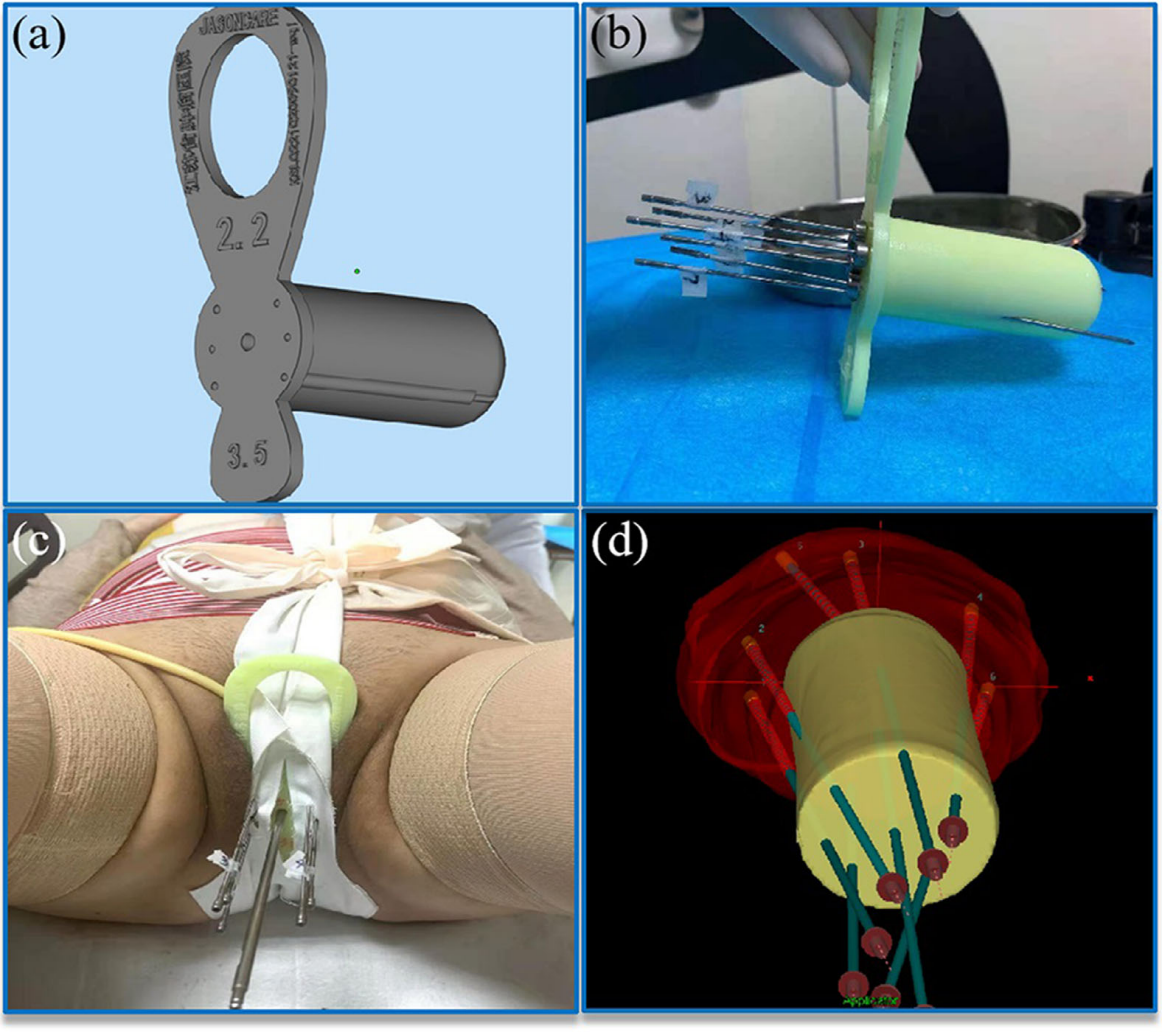
(a) Reconstruction of the template in the Mimics three‐dimensional (3D) modeling system based on the preplan. (b) 3D‐printed individualized template with inserted needles and the limiting stopper. (c) Immobilization of the template after completion of the implantation. (d) 3D model after reconstruction of the tumor target volume and needle passage. Red refers to the target volume, and yellow refers to the main part of the template.

Transverse CT images with the constructed intracavitary applicator and the virtual interstitial needles were sent to the Mimics 3D modeling system (Materialise NV, Leuven, Belgium) to establish the individual 3D template model (Figure 1a). The modeling file was transferred to the 3D‐printing machine (EP‐A650) to prepare the template using a stereo lithography appearance. Usually, a few hours are needed to complete the printing process, after which the template is sent to the sterilizing room in preparation for the clinical application. During the treatment, the 3D template can be redesigned through the procedure described above if the tumor topography has changed to a marked degree or if the doses of the HR CTV and OARs have not met the requirements.

### 2.3 Treatment process

The preparation process before insertion was the same as that followed for the preplan for all patients in both groups. Topical 10% lidocaine spray was used on the vaginal mucosa; afterward, 3 ml of 2% lidocaine was bilaterally injected into the para‐cervical region for local anesthesia. In the 3D template group, the individual template was implanted after the insertion of the intracavitary applicator. Then, the metal interstitial needles were assembled with limiting stoppers according to the recorded insertion depth and implanted into the reserved passage in the template. Patients were immobilized with a cotton tape designed by our research group after the operation process (Figure 1C). In the freehand group, the gynecologist decided the path of the intracavitary applicator and interstitial needles based on previous magnetic resonance images and gynecological examination and operated with CT guidance. When the entire implantation process was completed, 100 ml of saline was infused into the bladder. The CT scan used for the clinical plan design and structure delineation was performed in the same manner as that for the preplan procedure. The treatment plan was created by reconstructing the applicator and needles, inverse planning the dwell position and iterative manual optimization of the dwell position and time. The requirement of the treatment planning was that 90% of the HR CTV received 100% of the prescribed dose (6.5 Gy per fraction). After approval by a gynecologist and senior physicist, the treatment plan was exported to the afterloader to be implemented by the therapist.

### 2.4 Statistical methods

The target volume, dosimetry parameters, and insertion depth of the preplan and treatment plan were recorded. CT scan time and frequency of the two groups were also recorded and compared. The parameters of the dose volume histogram (DVH) included the dose delivered to 90%, 98%, and 50% of the HR CTV (D_90_, D_98_, and D_50_, respectively); the volume of the HR CTV receiving 200% of the prescribed dose (V_200_); and the maximal dose delivered to at least 2 cc of the rectum, bladder, bowel, and sigmoid (D_2cc_). The comparison of the insertion depth between the preplan and treatment plan was performed using paired‐sample *t*‐tests, while the DVH parameters were examined using paired‐sample nonparametric tests. The differences between the 3D‐printed template group and the freehand group were evaluated using independent‐sample nonparametric tests. All analyses were performed with SPSS 26 (IBM Inc., Armonk, NY, USA) at a significance level of 5%.

## 3 RESULTS

**FIGURE 2:**
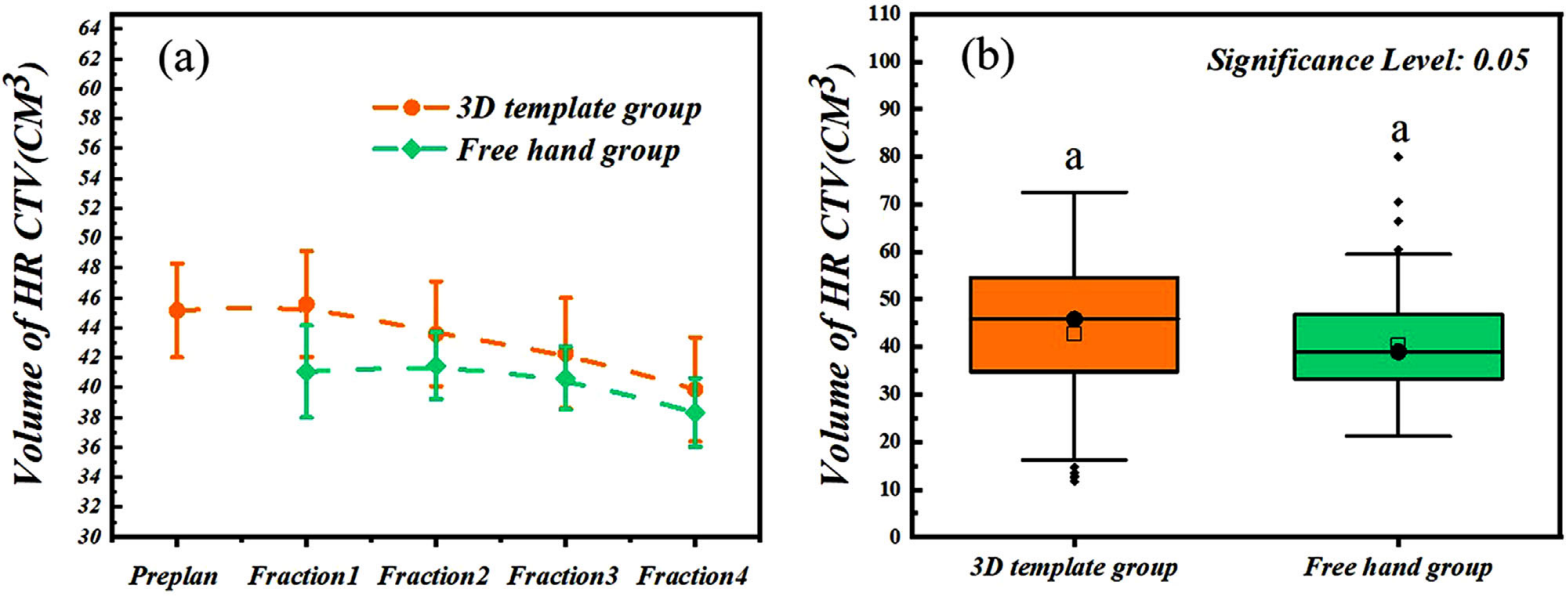
(a) High‐risk clinical target volume (HR CTV) volume of preplanning and subsequent treatment fractions (Fractions 1–4) for patients in two groups (Freehand group without the preplan). (b) Boxplots of the HR CTV volume at treatment fractions in two groups. Statistical significance is denoted by different letters, the same letters meant there was no significant difference between the two groups (*p* = 0.174).

Forty‐three patients all finished the treatment, two patients from the 3D template group and one from the freehand group changed to intracavitary brachytherapy due to the vessel bleeding in the first fraction. There was no perforation of the bladder or bowel for the 3D template group, while two patients in the freehand group were inserted into the bladder and were adjusted immediately. There was no statistical difference in the target volume of the two groups, respectively 43.10 ± 14.99 and 40.65 ± 11.25 (Table 1). The falling rate of target volume was more pronounced for patients in the 3D template group than in the freehand group (Figure 2a). There was a modest decrease in the HR CTV volume that can be observed from fractions 1 to 4 in the 3D template group, while it only appeared from fractions 3 to 4 in the freehand group. There was no obvious change between the preplan and fraction 1 for the 3D template group, which was meaningful for the guidance of the printed template.

The comparison of preplan with every treatment fraction is shown in Table 2. The path of the needles was controlled accurately because of the utilization of the template. The insertion depth in fractions 1 and 4 was a little shorter compared with preplan, while no significant difference was observed in fractions 2 and 3. None of the DVH parameters of fractions 1–3, nor the D_2cc_ of bladder and bowel at fraction 4 were barely changed compared with the preplan. The D_90_ and D_98_ of HR CTV in fraction 4 were increased compared with the preplan, and the D_2cc_ of the rectum and sigmoid were a little different from preplan.

**TABLE 2:** Insertion depth and dose‐volume histogram (DVH) parameters of preplan comparing with the treatment plan

	**Preplan**	**Fraction 1**	**Fraction 2**	**Fraction 3**	**Fraction 4**
Insertion depth(cm)	2.94 ± 1.61	2.69 ± 1.50	2.81 ± 1.56	2.73 ± 1.59	2.68 ± 1.62
***p*‐Value**	‐	0.001	0.166	0.055	0.044
HR CTV D_90_ (Gy)	6.55 (6.51–6.76)	6.59 (6.50–6.93)	6.76 (6.55–7.02)	6.75 (6.53–6.90)	6.83 (6.58–7.00)
***p*‐Value**	‐	0.775	0.145	0.230	0.034
HR CTV D_98_ (Gy)	5.28 (5.00–5.43)	5.45 (5.05–5.77)	5.60 (5.34–5.81)	5.51 (5.21–5.79)	5.64 (5.29–5.87)
***p*‐Value**	‐	0.236	0.052	0.103	0.009
Rectum D_2cc_ (Gy)	3.95 (2.62–4.54)	3.43 (2.77–3.98)	3.13 (2.21–3.78)	3.50 (2.43–4.06)	3.33 (2.56–3.74)
***p*‐Value**	‐	0.092	0.052	0.189	0.046
Bladder D_2cc_ (Gy)	4.66 (3.84–5.00)	4.52 (4.31–5.08)	4.53 (4.13–4.99)	4.38 (4.03–5.09)	4.64 (4.00–4.85)
***p*‐Value**	‐	0.909	0.864	0.627	0.530
Bowel D_2cc_ (Gy)	3.81(2.51–4.33)	2.95(2.56–4.38)	3.31(3.14–4.02)	3.65(2.53–4.25)	3.07(2.54–3.89)
***p*‐Value**	‐	0.253	0.689	0.265	0.116
Sigmoid D_2cc_ (Gy)	2.65(2.26–4.19)	3.91(2.66–4.41)	3.92(3.11–4.37)	3.62(3.00–4.15)	3.81(3.11–4.27)
***p*‐Value**	‐	0.123	0.072	0.278	0.049

Insertion depth values are expressed in the mean values ± standard deviations. DVH parameters values are expressed in the median with lower and upper quartiles indicating the range.

**FIGURE 3:**
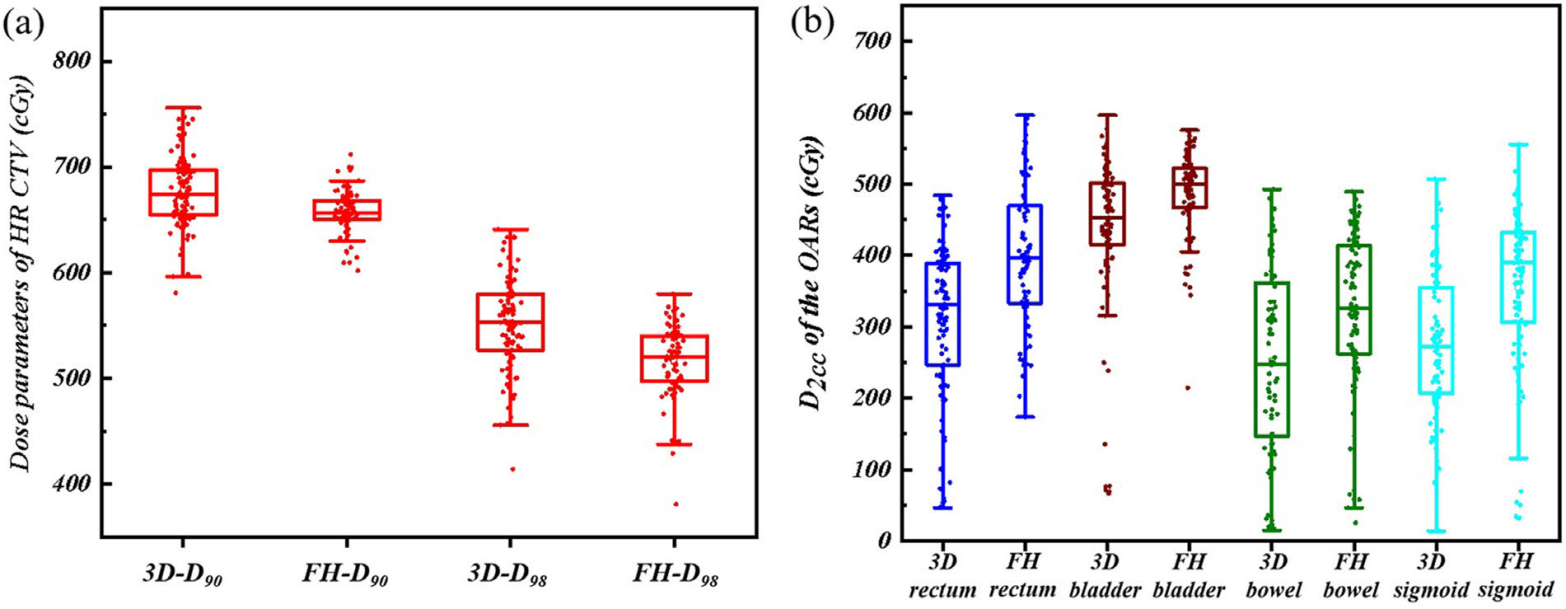
(a) Boxplots of the D_90_ and D_98_ of the high‐risk clinical target volume (HR CTV) in two groups. (b) Boxplots of the D_2cc_ of organs at risk (OARs) in two groups. (3D indicated the 3D template group, and FH indicated the freehand group.)

**FIGURE 4:**
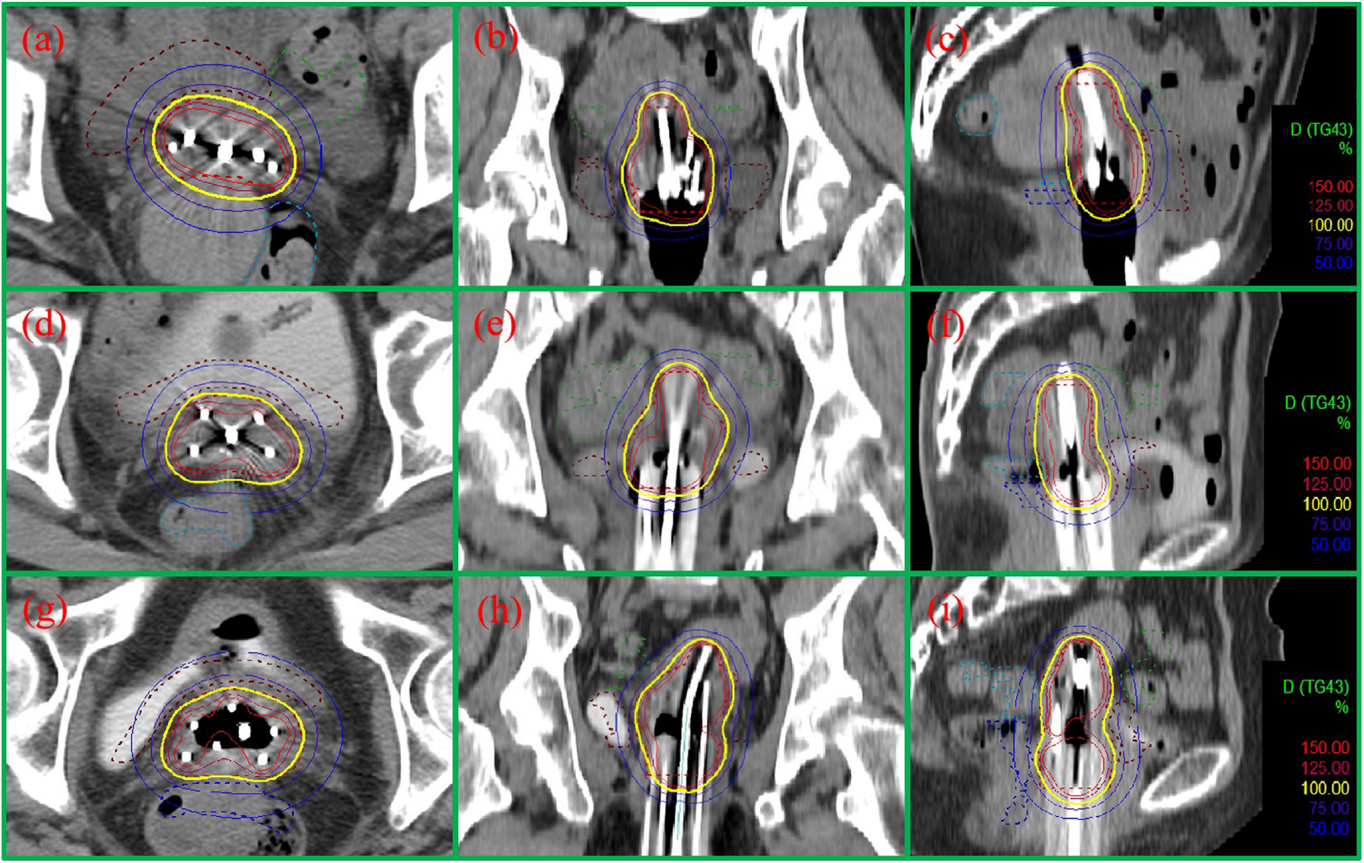
Dose distribution of treatment plans. (a–c) Patient from the freehand group treated with 4 needles. (D‐F). Patient from the 3D template group treated with 4 needles. (g–i) Patient from the 3D template group treated with six needles. Contours: red dashed line indicated high‐risk clinical target volume (HR CTV); dark blue dashed line indicated rectum; green dashed line indicated bowel; blue dashed line indicated sigmoid; brown dashed line indicated bladder.

As shown in Table 3, the D_90_ and D_98_ of HR CTV in the 3D template group were statistically higher than those in the freehand group (*p* < 0.01), while the D_50_ of HR CTV was a little lower than that in the freehand group (*p* = 0.045), and the V_200%_ of HR CTV displayed no statistic difference (*p* = 0.106). The D_2cc_ of the rectum, bladder, bowel, and sigmoid in the 3D template group were all lower than those in the freehand group (*p* < 0.01), which also can be distinctly observed in Figure 3. Typical plans from both groups were shown in Figure 4.

**TABLE 3:** Parameters of the 3D template group compared with the freehand group

	**3D template group**	**Freehand group**	***p*‐value**
HR CTV D_90_ (Gy)	6.74 (6.55–6.97)	6.57 (6.50–6.68)	<0.01
HR CTV D_98_ (Gy)	5.53 (5.27–5.79)	5.20 (4.97–5.40)	<0.01
HR CTV D_50_ (Gy)	10.71 (10.27–11.38)	10.91 (10.54–11.45)	0.045
HR CTV V_200%_ (cm3)	18.59 (14.89–22.15)	17.20 (14.17–19.84)	0.106
Rectum D_2cc_ (Gy)	3.32 (2.47–3.89)	3.97 (3.33–4.71)	<0.01
Bladder D_2cc_ (Gy)	4.53 (4.15–5.02)	5.01 (4.67–5.22)	<0.01
Bowel D_2cc_ (Gy)	2.48 (1.47–3.64)	3.26 (2.62–4.13)	<0.01
Sigmoid D_2cc_ (Gy)	2.72 (2.06–3.58)	3.89 (3.06–4.33)	<0.01
Insertion time (min)	9.90 (8.65–12.60)	19.15 (17.75–23.55)	<0.01
CT scan frequency	2 (1–2)	4 (3–4)	<0.01

All values are expressed in the median with lower and upper quartiles indicating the range.

## 4 DISCUSSION

Usually, major changes in the topography of the target volume and OARs occur after external irradiation and chemotherapy lasting 5 weeks.^22^, ^23^ Furthermore, the anatomy is distorted because of the intervention of the template and applicator, making preplanning necessary to achieve optimal needle track and isodose distribution. Several articles have studied the use of a 3D‐printed template for particle therapy,^24^, ^25^, ^26^ proposing the superiority of exact orientation and localization of inserted needles and considering it a result‐oriented and accurate auxiliary tool in brachytherapy. In this study, we utilized an offline preplan based on CT images 1 week before the treatment to design and print the template. Although an additional procedure was needed for 3D‐printed template treatment, more benefits can be obtained from its clinical application. First, owing to the inversely designed angle and depth of interstitial needles from the virtual plan, this method can reduce the CT scanning time required to guide the implant process, as well as the total time required for insertion. Second, it can reduce the risk of side effects, such as local bleeding and perforation of the bladder and bowel caused by unexpected needle positioning. Third, it can reduce the discomfort of patients during the implantation compared to online preplanning or free insertion. Finally, it is beneficial for the promotion of IS/IC therapy owing to less dependence on the skills and experience of the operators, which play a critical role in conventional insertion therapy.

The volume and topography of the target and OARs in the preplan should be consistent with the first treatment fraction to ensure the effectiveness of template guidance. As shown in Figure 2, the volume of the HR CTV barely changed from the preplan to the first fraction, but the depth of the needles in fraction 1 was a little shorter compared to that in the preplan (Table 2). There were no significant differences in the dose parameters of the HR CTV and OARs caused by a change in the insertion depth. However, the D_2cc_ of the bladder and sigmoid were a little higher in fraction 1 than in the preplan in the upper and lower quartiles. Inadequate insertion may be the main reason for the change in the OARs on the premise of achieving sufficient dose coverage of the HR CTV. In both fractions 2 and 3, there were no significant dosimetry changes in the HR CTV or OARs, and the insertion depth also approached that in the preplan. Nevertheless, all parameters except the D_2cc_ of the bladder and bowel in fraction 4 had differences compared to those in the preplan caused by a reduction in the HR CTV volume and topography change after three fractions of brachytherapy. However, the difference in fraction 4 was favorable to the treatment plan, as the D_90_ and D_98_ of the HR CTV obviously increased. The template used in this study was not regulated throughout the therapy. According to the results of parameter changes between the clinical treatment and virtual plan, we deemed that more dosimetry gain can be achieved if we adjust the template in the middle of the therapy (fraction 3) based on the previous fraction data, which can be considered adaptive brachytherapy for the treatment that follows. The average needle insertion depth in a previous study was 25 mm,^27^ consistent with our results. This study did not examine the influence of structure topography on dose changes; we plan to study this aspect in the future.

Oblique needles were used to increase the area covered by the residual tumor at the pelvic wall or in the distal parametria. The number of interstitial needles used in our study was 4–7, of which there were at least two oblique needles. For the previous standard transperineal templates, such as the Martinez Universal Perineal Interstitial Template,^28^ the needle path to the tumor was long, making it difficult to ensure the security and accuracy of the implanting process. Moreover, the risk of complications may increase with the increasing number of interstitial needles, and patients experience significant pain during the lengthy insertion process. The purpose of using our designed template is to reduce the time and pain of surgery by optimizing the path of the needles in an offline virtual plan and decreasing the number of inserted needles as much as possible, rendering it more suitable for application to medical conditions seen in China owing to its large population base.

According to the recommendation of the American Brachytherapy Society,^29^ the biologically equivalent dose in 2 Gy fractions (EQD_2_) of D_90_ for the HR CTV should exceed 80 Gy after external irradiation and brachytherapy (α/β = 10 Gy for HR CTV and 3 Gy for OARs). Previous studies have found that tumor control rates were higher than 90% for all patients when D_90_ for the HR CTV exceeded 86 Gy.^30^, ^31^ All patients in this study met the requirements. Both the D_90_ and D_98_ for the HR CTV in the 3D template group were higher than those in the freehand group (Figure 3a). The V_200%_ of the HR CTV was not significantly different, possibly because intracavitary applicators were utilized in both groups. The constraint of the D_2cc_ of the rectum and sigmoid was <75 Gy, and that for the bladder was <90 Gy. In this study, the dose of the OARs was not out of bounds, and the D_2cc_ of the OARs in the 3D template group was statistically lower than that in the freehand group. As shown in Figure 4a, the distance between the interstitial needles in the freehand group was usually very close, which was not in conformity with the principles of brachytherapy. We found that it was difficult for the gynecologist to insert the needles uniformly and tautologically into the ideal position in the freehand group at every fraction. In addition, there was not sufficient visual operation space to insert more needles in free implantation, and that was why the number in the 3D template group was a little higher than that in the freehand group. With the reserved pathway in the template for needles, we could achieve repeatable and appropriate distribution of needles in the 3D template group, which also resulted in better dosimetry distribution (Figure 4D–I).

There are some inadequacies in this study. First, the initial recording HR CTV volume of the 3D template group was much larger than that of the freehand, which is difficult to evaluate how the 3D template could improve the plan quality when HR CTV are about the same. Thus, the template in the HR CTV was delineated and subtracted. The actual volume of HR CTV in the 3D template group was 43.10 ± 14.99, which had no statistical difference from that in the freehand group (40.65 ± 11.25) (*p* = 0.174). Second, the difference in patient age. The principle of patients chosen was primarily the pathological stage and the topography of the tumor rather than the age in this study. However, the main outcomes in our study were the dosimetry characteristics of the patients, which were mainly relative to the size and topography of the HR CTV. Also, we have researched the influence of age on the outcomes of cervical cancer. In the study of Khalkhali et al.^32^ the diagnosis age less than 50 years with a mean survival time of 102.4 months was significantly different from 50 years and over (66.7months). Serur et al.^33^ had a study on age, substance abuse, and survival of patients with cervical cancer performed on 1173 patients, and the results showed that women over 70 years had a lower chance of survival compared to younger women. So, in our opinion, the difference in age in this research would not affect the results of the dose comparison between the two groups. This article presented the preliminary results of the clinical application of our designed 3D‐printed template. More patients and their survival outcomes need to be included in the future, and we could analyze follow‐up data by dividing them into groups depending on age.

Transrectal ultrasonography (US) is usually effective and feasible for guiding needle insertion because of the accurate location of interstitial needles and avoidance of rectum or bladder perforations. Sharma et al.^34^ used the transrectal US to guide needle implantation for 25 patients, and the insertion process lasted 50 min on average (number of needles, 13–23) with no perforation observed. Unfortunately, transrectal US equipment is not yet available at our center. Thus, we used CT scanning to guide the implantation procedure. We found that the implanting process was more convenient without excessive use of CT scanning (basically 1–2 times per fraction) with the use of the individual template. However, free needles were difficult to insert at an appropriate angle and depth, which needed additional CT scanning at a frequency of three to four times to verify the position. For freehand insertion, CT scanning frequency primarily depends on the total number of inserted needles. Although few freehand needles were used in this study, the insertion time for the freehand group was much longer than that for the 3D template group (Table 3). This study presented the preliminary results of the clinical application of our designed 3D‐printed template. More patients need to be included in future studies to support our results.

## 5 CONCLUSION

The preplan in this study was consistent with treatment plans, which is important to ensure the feasibility of applying a 3D‐printed template in brachytherapy. The 3D‐printed individual guidance template was an effective method in brachytherapy for LACC with convenient operation, improved reproducibility, accurate localization, and a lower number of needles used. However, follow‐up, local control rates, and survival rates should be analyzed to support the advantage of this technology in the future.

## AUTHOR CONTRIBUTIONS

Bin Zhang: methodology, investigation, data analysis, writing original draft, funding acquisition;

Siyu Zhang: data collection, investigation, data analysis;

Li Sun: data collection, data validation;

Yaqin Wu: conceptualization, funding acquisition, supervision, review, and editing;

Yongqin Yang: conceptualization, supervision, review, and editing.

## CONFLICT OF INTEREST

The authors declare that they have no conflict of interest.

## Data Availability

The data that support the findings of this study are available on request from the corresponding author. The data are not publicly available due to privacy or ethical restrictions.
